# Degradation of Diuron by *Phanerochaete chrysosporium*: Role of Ligninolytic Enzymes and Cytochrome P450

**DOI:** 10.1155/2013/251354

**Published:** 2013-12-31

**Authors:** Jaqueline da Silva Coelho-Moreira, Adelar Bracht, Aline Cristine da Silva de Souza, Roselene Ferreira Oliveira, Anacharis Babeto de Sá-Nakanishi, Cristina Giatti Marques de Souza, Rosane Marina Peralta

**Affiliations:** Department of Biochemistry, State University of Maringa, 87020-900 Maringá, PR, Brazil

## Abstract

The white-rot fungus *Phanerochaete chrysosporium* was investigated for its capacity to degrade the herbicide diuron in liquid stationary cultures. The presence of diuron increased the production of lignin peroxidase in relation to control cultures but only barely affected the production of manganese peroxidase. The herbicide at the concentration of 7 **μ**g/mL did not cause any reduction in the biomass production and it was almost completely removed after 10 days. Concomitantly with the removal of diuron, two metabolites, DCPMU [1-(3,4-dichlorophenyl)-3-methylurea] and DCPU [(3,4-dichlorophenyl)urea], were detected in the culture medium at the concentrations of 0.74 **μ**g/mL and 0.06 **μ**g/mL, respectively. Crude extracellular ligninolytic enzymes were not efficient in the *in vitro* degradation of diuron. In addition, 1-aminobenzotriazole (ABT), a cytochrome P450 inhibitor, significantly inhibited both diuron degradation and metabolites production. Significant reduction in the toxicity evaluated by the *Lactuca sativa* L. bioassay was observed in the cultures after 10 days of cultivation. In conclusion, *P. chrysosporium* can efficiently metabolize diuron without the accumulation of toxic products.

## 1. Introduction 

Agricultural practices are among the main activities responsible for the release of hazardous chemicals into the environment [1]. Among these chemicals, the pesticides (fungicides, herbicides and insecticides) have been used for decades without any control, resulting in a strong contamination of water, air, and foods as well as in the development of pesticide resistant organisms. This problem became more serious during the last years resulting in high risks to human health.

Herbicides are the main class of pesticides used extensively in home gardens and farms all over the world [2]. Diuron is a phenylurea herbicide applied to a wide variety of crops, especially sugar-cane cultures. The compound acts in photosynthetic organisms by blocking electron transport in photosystem II, thus inhibiting photosynthesis. In the environment diuron can be transformed abiotically via hydrolysis and photodegradation reactions, but under natural conditions these reactions occur at very low rates [3]. Due to this, diuron is known as a potential water contaminant being frequently detected at concentrations ranging from 2.7 *μ*g/mL to 2849 *μ*g/mL in surface water and 0.34 to 5.37 *μ*g/mL in groundwater [4]. The dissipation of diuron from the environment is thus mainly due to biotic processes, the aerobic microbial metabolism being the major form of diuron transformation [3, 5]. The main reactions involved are N-demethylation and hydrolysis of the amide bond. In most cases, mono- and didemethylated compounds and 3,4-dichloroaniline appear as the main products of microbial metabolism [3, 6]. These metabolites accumulate in the environment and some of them are more toxic than diuron [7].

**Figure 1:**
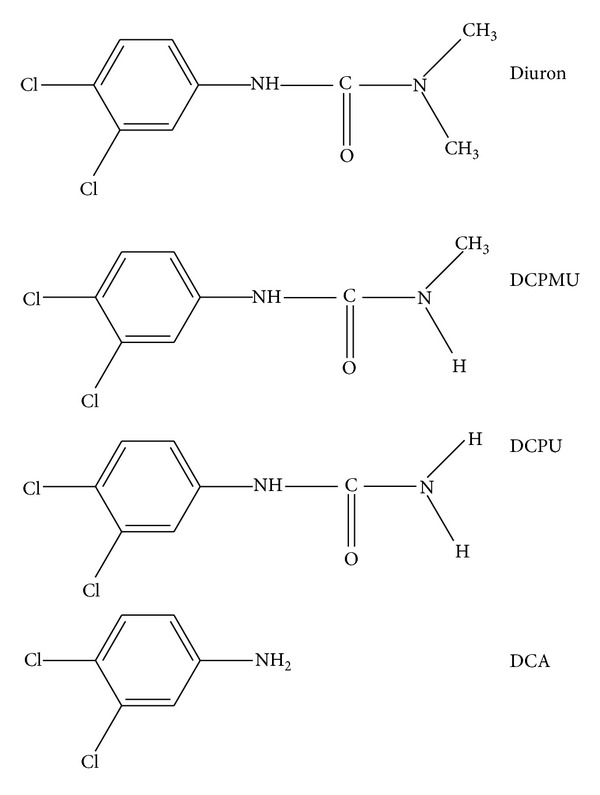
Chemical structure of diuron and its main metabolites DCPMU, DCPU, and DCA.

The aerobic biodegradation pathway for diuron is well established (Figure 1). It proceeds by successive demethylation steps to form DCPMU [1-(3,4-dichlorophenyl)-3-methylurea], DCPU [1-(3,4-dichlorophenyl)urea], and 3,4 DCA (3,4-dichloroaniline). Several studies describe the capability of soil fungi to degrade diuron with accumulation of these N-demethylated metabolites [7–9]. Among white-rot fungi, the degradation of diuron is generally attributed to the action of extracellular enzymes, the lignin-modifying enzymes typically produced by these fungi [10–13]. However, the products generated in the degradation have not yet been characterized and no efforts have been done to evaluate their toxicity.

The model of white-rot fungus for many bioremediation studies is *Phanerochaete chrysosporium* [14]. Its ability to degrade pollutants appears to be related especially to the production of lignin peroxidase and manganese peroxidase, two lignin-modifying enzymes generally expressed under nitrogen-limited culture conditions [15], as well as to the intracellular cytochrome P450 system [16]. The transformation of diuron by *P. chrysosporium* in liquid cultures has already been documented in both stationary and shaken conditions [11, 12]. Stationary cultures are advantageous over shaken cultures because they work without mechanical energy requirements, thus increasing the feasibility of the technique for application in large scale treatment of wastewater. The metabolic processing of diuron by *P. chrysosporium* is still not completely clarified specially with respect to the metabolites that are produced and the role of cytochrome P450 in the degradation. Taking this into consideration, the objectives of this work were to study the removal of diuron from liquid cultures of *P. chrysosporium* with special interest in the role of cytochrome P450 and identification of demethylated metabolites. Attempts were also done to compare the toxicity of diuron metabolites with the parent molecule.

## 2. Materials and Methods

### 2.1. Chemicals

The enzymatic substrates, diuron (≥98%), DCPMU, DCPU 3,4-DCA (3,4-dichloroaniline), and ABT (1-aminobenzotriazole) were obtained from Sigma Chemical Corp. (St Louis, MO). Stock solutions of diuron, DCPMU, DCPU, 3,4-DCA, and ABT were prepared by dissolving standards in dimethyl sulfoxide **(**DMSO), filtering through a millipore membrane (0.45 mm), and storing at 4°C. PDA was obtained from Difco Laboratories (Detroit, MI). The solvents used in the HPLC analyses were of chromatographic grade and all other reagents were of analytical grade.

### 2.2. Microorganism and Inoculum

*Phanerochaete chrysosporium* was obtained from the André Tosello Foundation (ATCC 24725) and cultured on potato dextrose agar (PDA) for 7 days at 28°C. Mycelial plugs measuring 15 mm in diameter were made and used as inoculum for liquid cultures.

### 2.3. Culture Conditions

The experiments were performed in liquid medium under stationary conditions at 28°C in the dark. *P. chrysosporium* was cultivated in 125 mL Erlenmeyer flasks using three mycelial disks on PDA plates (approximately 15 mm in diameter) for up to 12 days. Each flask contained 25 mL of a medium prepared with a mineral solution without nitrogen source [17] containing 1.2 mmol/L ammonium tartrate in order to obtain a nitrogen-limited medium, that is favorable to ligninolytic enzyme production. Additionally, to induce the ligninolytic enzymes, a corn cob extract rich in phenolic compounds was used. For preparation of the extract, an aqueous suspension containing 3% corn cob powder (w/v) was boiled for 5 minutes and filtered through Whatman filter paper number 1 to retain the residues and to avoid diuron adsorption on the insoluble corn cob. Afterwards, the mineral solution was prepared using the corn cob extract enriched with 1% glucose as carbon source. The medium was previously sterilized by autoclaving at 121°C for 15 min.

### 2.4. Effect of Diuron in the Biomass Production of *P. chrysosporium*

Increasing amounts of diuron dissolved in DMSO (30 to 100 *μ*mol/L) were added to the liquid medium at the beginning of the cultivation. The DMSO volume added was not superior to 30 *μ*L in order to not affect the fungal metabolism. After 10 days the cultures were interrupted by filtration and the mycelia were washed three times with distilled water and dried to constant weight at 50°C.

### 2.5. Time Course of Enzyme Production and Diuron Degradation

To evaluate the capability of *P. chrysosporium* liquid cultures to degrade diuron, each culture flask received a volume of a diuron stock solution in DMSO (10 mg/mL) to give a final concentration of 30 *μ*mol/L (7 *μ*g/mL). The flasks were then agitated to mix the herbicide solution and inoculated with the fungus. Two types of controls were conducted in parallel. In the first, the fungus was inoculated in liquid medium without herbicide. The second control consisted of sterile medium containing the same amount of diuron (abiotic control). All flasks were incubated for up to 12 days. At periodic intervals, the cultures were interrupted by filtration, and the culture extracts were used to evaluate the lignin-modifying enzymes, lignin peroxidase and manganese peroxidase, as well as the residual diuron and its metabolites by high-performance liquid chromatography (HPLC).

### 2.6. Lignin-Modifying Enzyme Assays

Manganese peroxidase (Mn peroxidase) activity was assayed by the oxidation of 1 mmol/L MnSO_4_ in 50 mmol/L sodium malonate buffer pH 4.5, in the presence of 0.1 mmol/L H_2_O_2_. Manganic ions (Mn^3+^) form a complex with malonate, which absorbs at 270 nm (*ε*_270_ = 11,590 M^−1^ cm^−1^) [18]. Lignin peroxidase activity was determined by spectrophotometric measurement at 310 nm of the H_2_O_2_-dependent veratraldehyde formation from 0.4 mmol/L veratryl alcohol in 0.1 mol/L tartrate buffer of pH 3.0 [19]. Laccase activity was assayed by measuring the oxidation of 2,7′-azinobis [3-ethylbenzothiazolone-6-sulfonic acid] diammonium salt (ABTS) [20]. The enzymatic activities were expressed as International Units (U), defined as the amount of enzyme required to produce 1 *μ*mol product per min at 40°C.

### 2.7. Analysis of Residual Diuron and Identification of Diuron Metabolites

For extraction of diuron and its metabolites from the mycelia, a volume of 6 mL of acetonitrile was added to the cells and the mixture was maintained for 2 h under agitation at 130 rpm. The mycelial extracts were obtained after centrifugation at 5,000 rpm for 15 min at 4°C. The concentrations of diuron and its metabolites in the culture media and in the mycelial extracts were determined using an HPLC system (Shimadzu, Tokyo) with an LC-20AT Shimadzu system controller, Shimadzu SPD-20 A UV-VIS detector, equipped with a reversed Shimpack C18 column (4.6 × 250 mm), maintained at 40°C. The mobile phase consisted of water (solvent A) and acetonitrile (solvent B) at a flow rate of 0.8 mL/min. The gradient program started with 25% acetonitrile increasing to 85% in 15 minutes. The column was equilibrated for 10 minutes before the next injection. The UV detection was made at 245 nm and the injection volume was 20 *μ*L. All samples in triplicate were filtered through a membrane filter (0.45 *μ*m) before injection. The concentrations of diuron and its metabolites were determined using calibration curves constructed with peak areas of authentic standards (diuron, DCPMU, DCPU, and DCA). The identification of the compounds was based on their respective retention times and on the fortification of the samples with standards. Under the conditions employed, diuron was eluted at 11.6 min, DCPMU at 10.6 min, DCPU at 9.5 min, and 3,4-DCA at 12.1 min.

### 2.8. Cytochrome P450 Inhibition Studies

The cytochrome P450 inhibitor, 1-aminobenzotriazole (ABT), was added to *P. chrysosporium* cultures at the start of the cultivation to obtain a final concentration of 1 mmol/L. A diuron stock solution was added to a final concentration of 30 *μ*mol/L (7 *μ*g/mL). Control cultures without cytochrome P450 inhibitor were incubated in parallel. The cultures were inoculated and incubated as described above. The diuron degradation in the presence and absence of ABT was measured after 5 and 10 days of cultivation.

### 2.9. *In Vitro* Degradation of Diuron by Extracellular *P. chrysosporium* Crude Enzymes

The capability of extracellular *P. chrysosporium* crude enzymes to degrade diuron was evaluated using 7-day culture filtrates according to the protocol described previously [21]. The reaction mixture (1 mL) contained (final concentration) 7 *μ*g/mL diuron, 40 U/L crude lignin peroxidase, 50 U/L manganese peroxidase, and 0.1 mmol/L H_2_O_2_ in tartrate buffer 0.1 mol/L, pH 3.0. The effects of adding 0.5 mmol/L MnSO_4_ and 0.5 mmol/L veratryl alcohol were tested separately or in combination. All reactions were conducted in sterile tubes at room temperature. After 24 h, the reaction mixtures were filtrated with a membrane filter (0.45 *μ*m) before the HPLC analyses.

### 2.10. Toxicological Test

The toxicity assessment of crude culture filtrates and abiotic control samples was conducted using lettuce seeds (*Lactuca sativa*). The crude filtrates were obtained from 10-day cultures using an initial concentration of diuron of 7 *μ*g/mL. The bioassay was conducted with five dilutions of each sample in water (v/v) (100, 80, 50, 20, and 10%). Twenty seeds were placed in 90 mm diameter Petri dishes containing filter paper saturated with 3 mL of different dilutions of samples or water (control) [22]. After 5 days, the number of germinated seeds was counted and the lengths of the radicles and hypocotyls were measured. The data were represented as absolute germination percentage and relative growth compared to the control as follows:

(1) $$\begin{array}{c} \text{Absolute}\;\text{germination}\left(\%\right)=\frac{\text{GS}}{\text{TS}}\times 100,\\ \text{Relative}\;\text{growth}\;\text{of}\;\text{the}\;\text{radicle}/\text{hypocotyl}\left(\%\right)=\frac{\text{LS}}{\text{LC}}\times 100, \end{array}$$

where GS = number of germinated seeds, TS = number of total seeds, LS = average length of the sample, and LC = average length of the control.

To calculate the dose that produced 50% inhibition of germination or growth (LD_50_), the coefficient of inhibition *I* (%) for each parameter was calculated as follows:

(2) $$\begin{array}{c} I\;{\left(\%\right)}_{\text{germination}}=\frac{\text{GSC}-\text{GSS}}{\text{GSC}}\times 100,\\ I\;{\left(\%\right)}_{\text{radicle}/\text{hypocotyl}\;\text{growth}}=\frac{\text{LC}-\text{LS}}{\text{LC}}\times 100, \end{array}$$

where GSC = number of germinated seeds in control and GSS = number of germinated seeds in sample.

## 3. Results

### 3.1. Effects of Diuron on Biomass Production and Production of Ligninolytic Enzymes

**Figure 2:**
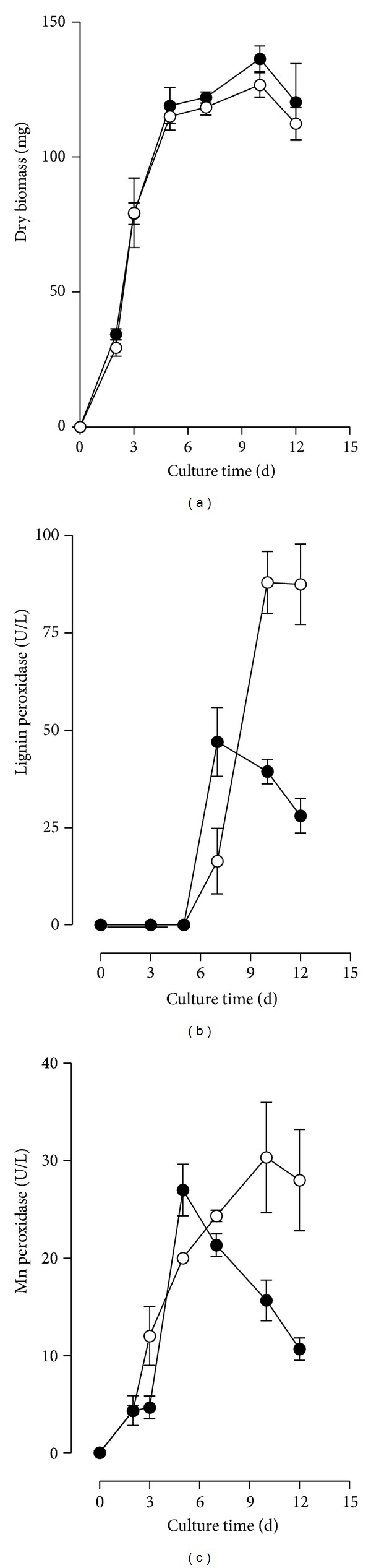
Effect of diuron in the biomass production (a), production of lignin peroxidase (b), and production of manganese peroxidase (c). Absence of diuron (Control cultures) (*⚫*); with 7 *μ*g/mL diuron (○).

Concentrations of diuron up to 80 *μ*mol/L (18.6 *μ*g/mL) only barely affected the fungal biomass produced after 10 days of cultivation when compared with the fungal biomass obtained in the absence of herbicide. An increase to 100 *μ*mol/L (23.2 *μ*g/mL) in the concentration of diuron resulted in high inhibition of the biomass production. At 7 *μ*g/mL of diuron, the growth curve was very similar to the growth curve in the absence of herbicide (Figure 2(a)). The addition of diuron increased the LiP activities from 47 U/L (at day 7) to 88 U/L (at day 10) (Figure 2(b)). The maximal Mn peroxidase activity was barely affected by the presence of diuron. After 5 days of cultivation, the levels of Mn peroxidase were 22.4 and 20.0 U/L in the presence and absence of diuron (Figure 2(c)). After 10 days of cultivation, the levels of Mn peroxidase found in the culture filtrates in the presence and absence of diuron were 29.4 and 15.4, respectively. Laccase activity was not detected under any condition with or without diuron.

### 3.2. Diuron Degradation Experiments

**Figure 3:**
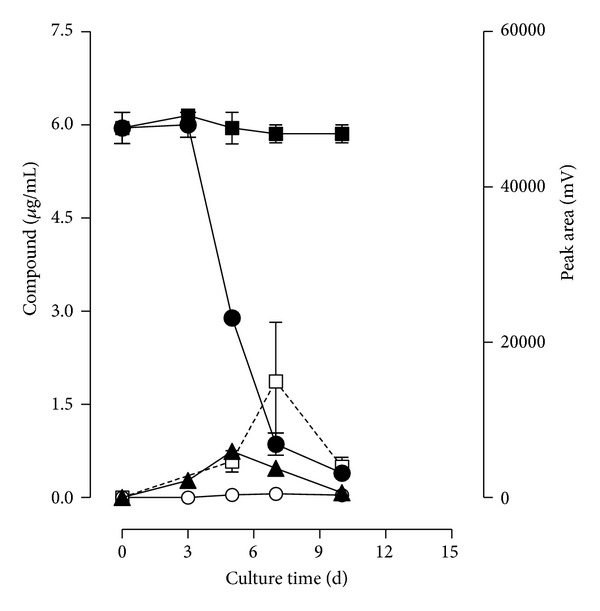
Time course of diuron degradation (*⚫*) and formation of DCPMU (▲), DCPU (○), and an unknown product (□) eluted at 15.2 min. Recovery of diuron (■) from the uninoculated control culture is also shown.

*P. chrysosporium* showed high rates of diuron removal in the corn cob liquid medium under the nitrogen-limited condition used in this work (Figure 3) and this fact seems to be associated with the mycelium growing phase. After 5 days of cultivation, the fungus was able to remove approximately 52% of diuron and at the end of the experiment the removal reached 94%. Concomitantly with the removal of diuron, the formation of diuron metabolites was detected, namely, DCPMU and DCPU. Identification of the metabolites was carried out by comparison of their retention times with those obtained by injecting standards under the same conditions as well as by spiking the samples with stock standard solutions. The concentration of DCPMU reached 0.74 *μ*g/mL at day 5 and decreased to 0.08 *μ*g/mL at the end of the experiment. Only traces of the metabolite DCPU were observed (less than 0.06 *μ*g/mL) during the whole cultivation. An unidentified peak (retention time of 15.2 min) was detected with a maximal area at day 7 and decreased until the end of the experiment. No 3,4-DCA production was detected in the *P. chrysosporium* cultures. All these compounds were not observed in cultures without diuron.

### 3.3. Effect of Cytochrome P450 Inhibitor on Fungal Metabolism

**Figure 4:**
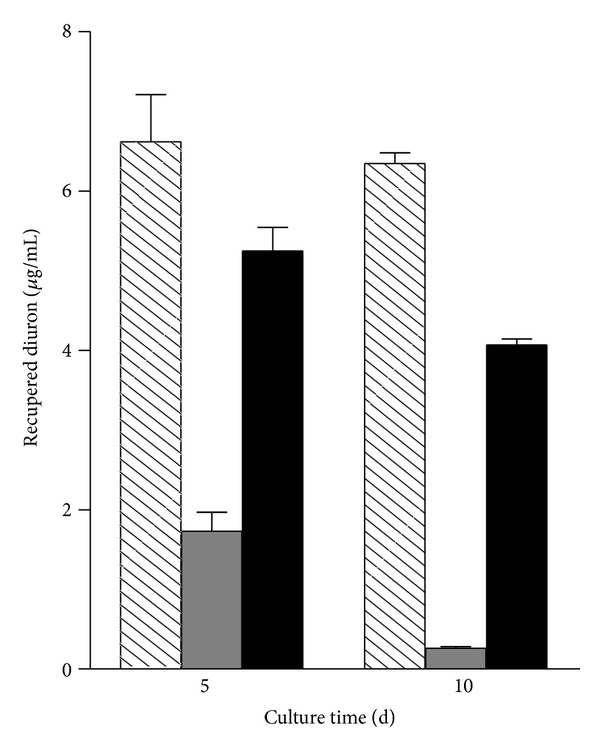
Effect of the cytochrome P450 inhibitor (ABT) on diuron degradation. Treatments: abiotic controls (striped bars), inhibitor-free control (grey bars), and cultures with ABT (black bars).

The involvement of the cytochrome P450 in the diuron degradation was examined by adding the cytochrome P450 inhibitor (ABT) to the cultures containing the herbicide at the beginning of the cultivation. The diuron degradation was clearly inhibited by the addition of 1 mmol/L ABT. As shown in Figure 4, in cultures where ABT was added, higher concentrations of diuron were observed, whereas in inhibitor-free cultures diuron was almost completely removed when compared to the uninoculated controls. The addition of ABT did not affect the fungal biomass production (not shown).

The production of N-demethylated metabolites was clearly affected by the presence of the cytochrome P450 inhibitor. The formation of the metabolite DCPMU was the most strongly inhibited. As shown in Table 1, in control cultures the levels of DCPMU decreased until the end of the experiments, suggesting that this compound was metabolized by the fungus. In contrast, in the presence of ABT this metabolite accumulated in the medium, as can be observed by the slight increase of its concentration at day 10, which indicates that the metabolization of DCPMU was also inhibited by ABT. The effect of ABT on the formation of the metabolite DCPU was less pronounced causing a slight decrease of its level when compared with the control.

The amounts of diuron and its metabolites in the culture filtrate and mycelial extract after 5 days of cultivation are shown in Table 2. It is noteworthy to mention that, in fractional terms, the amounts of DCPMU and DCPU in the mycelial extract exceeded those of the parent compound diuron by factors of 1.73 and 1.99, respectively. Furthermore, the total amount of diuron and its metabolites inside and outside of the cells corresponds to 61.6% of the amount of diuron added to the cultures. This indicates that a substantial fraction was removed. Unfortunately, chromatographic analyses of 10 d-mycelial extracts could not be evaluated considering the presence of interfering molecules, also present in the cell extracts obtained in the absence of diuron.

### 3.4. Degradation of Diuron by Crude Enzymatic Extract

The capability of crude enzymatic extracts to degrade diuron *in vitro* was tested under different conditions (Table 3). No significant differences (*P* < 0.05) were observed between the control and the treatments containing enzymes and mediators.

### 3.5. Toxicity Tests

The samples showed moderate toxic effects without dilution (100%) or when the samples were diluted by a factor of 1.25 (80%) (Table 4). Toxic effects of the samples were observed on the radicles and hypocotyl, such as reduction in size, necrosis, and fragility. In relation to the radicle growth, no reduction in the toxicity between treated and nontreated samples (abiotic control) was observed. In relation to the hypocotyl development, it was possible to observe a reduction in the toxicity after transformation of diuron by *P. chrysosporium*: while nontreated samples promoted an effective inhibition of the hypocotyl growth, treated samples allowed a better development of this structure, what demonstrates a detoxification of the medium by the fungal treatment. In these analyses, the inhibition coefficient allowed to calculate the LD_50_. In this case, the LD_50_ indicates the acute toxicity of the samples and its value represents the sample dilution (v/v) that produced 50% inhibition of germination or growth. The lower the LD_50_ value, the more toxic the sample. Therefore, it is possible to compare the toxicities of different samples. For radicle growth, the LD_50_ values were 15.4% and 58.7% for nontreated and treated samples, respectively. For hypocotyl growth, the LD_50_ was of 51.6% for nontreated and of 95.1% for treated samples. The highest LD_50_ values were achieved with treated samples, indicating that there is a smaller toxicity for 10-day-treated samples. Therefore, a detoxification process seems to have occurred in presence of the fungus.

## 4. Discussion

It is well known that *P. chrysosporium* possesses a great ability to degrade herbicides, for example, isoproturon [23], atrazine [24], propanil [11], bentazon [25], and also diuron [10]. Most studies have obtained the greatest degradation levels in solid state cultures, whereas in liquid medium the degradation efficiency seems to be smaller. In this study, the stationary liquid culture was used because it is specially suitable for wastewater treatment due to its simplicity and low cost, providing conditions for high removal of diuron and identification of the main metabolites.

The expression of ligninolytic enzymes by *P. chrysosporium* in the liquid system used in this work was favored by the limitation of nitrogen and by the presence of soluble corn cob phenolics. Different studies where the capability of *P. chrysosporium* to degrade herbicides was evaluated have suggested the participation of ligninolytic enzymes in the process based on a temporal coincidence between herbicide degradation and maximal enzyme activities [10, 23]. The participation of extracellular enzymes in the transformation of several herbicides by some white-rot fungi, including *P. chrysosporium,* was conclusively demonstrated by studies performed with purified enzymes and compiled in a recent review [2]. In the present work a temporal correlation between production of ligninolytic enzymes and initial diuron degradation was evidenced. However, no transformation of the herbicide was observed when the crude culture filtrates were incubated *in vitro* with diuron. These results indicate that the first steps of diuron transformation seem to be more associated with the mycelial components than with the extracellular enzymes. In relation to the increase in the activity of lignin peroxidase, this phenomenon is not necessarily related to the herbicide transformation, since this effect can be due to several alterations in cell physiology or in plasmatic membrane structure, which reflect the adaptation of the fungus to the polluted environment [26]. These considerations support the hypothesis that diuron degradation involves an intracellular enzymatic system and weaken the hypothesis of extracellular LiP and MnP participations in the initial reactions. Two other observations of the present work reinforce the hypothesis that the degradation of diuron started intracellularly: the considerable amounts of diuron, DCPMU, and DCPU found in fresh mycelia and the inhibition of degradation caused by ABT, a cytochrome P450 inhibitor.

The participation of an intracellular enzymatic mechanism, represented mainly by cytochrome P450, in the degradation of different xenobiotics has been extensively considered in the last years. Purification of fungal cytochrome P450, in order to obtain conclusive data, has been accomplished in only a few studies, due to the difficulties in keeping the activation of the enzymes during microsome preparation. Hence, most conclusions were drawn from the results of indirect experiments consisting in the addition of specific cytochrome P450 inhibitors to the culture medium, such as piperonyl butoxide and 1-aminobenzotriazole, the same strategy used in the present work. Two recent studies reinforced the importance of this system in the *P. chrysosporium* degradation of pentachlorophenol [27] and phenanthrene [28]. In the latter study strong evidence has been presented for the participation of cytochrome P450 monooxygenases in anthracene metabolism by *P. chrysosporium.*

The chromatographic analysis demonstrated that diuron was effectively transformed by the fungus. DCPMU was the major N-demethylated metabolite identified and did not accumulate in the medium, suggesting that this compound was further degraded by the fungus. Another metabolite appeared in the medium but, unfortunately, it could not be identified by the methods employed in the present work.

Besides *P. chrysosporium* [10–12], other white-rot species, such as *Bjerkandera adusta* and *Trametes versicolor* [12, 13] and *Pleurotus ostreatus* [11, 12], have also been reported to degrade diuron in liquid cultures. In these studies no efforts were made to identify the transformation products. Our results show that diuron transformation by *P. chrysosporium* appears to be similar to that described for some soil fungi, that is, by successive N-demethylation, but with no formation and accumulation of 3,4-DCA, considered to be the most toxic and persistent metabolite of diuron [3].

Although diuron has been efficiently degraded in fungal cultures, conclusions drawn from this kind of experiments should not be extrapolated straightforwardly to nature because the biological degradation is frequently incomplete and may produce metabolites that are more toxic than the initial compound. The toxicological tests using lettuce seeds as bioindicators represent an effort to compare the toxicity of treated and nontreated diuron samples and thus to assess the effectiveness of *P. chrysosporium* in reducing environmental contamination [22]. Previous studies showed that diuron is transformed by fungal cultures producing N-demethylated metabolites with a higher toxicity than diuron [7, 29]. In such works, the toxicity was evaluated according to the Microtox test after isolation and concentration of the metabolites. The present work was conducted to evaluate the general toxicity of the culture extracts after just a single centrifugation step, used in order to clean the samples. The results showed a significant decrease in the toxicity of culture extracts at the end of the fungal treatment, which coincided with the decrease of the medium concentrations of both diuron and its metabolites due to the degradation processes and also to the uptake of these compounds into the cells.

## 5. Conclusion

The present study emphasizes the capability of *P. chrysosporium* to degrade the herbicide diuron. The fungus was able to remove 94% of the herbicide after 10 days of cultivation with no apparent accumulation of toxic products. This work complements the results obtained by other authors which demonstrate that demethylation at the terminal nitrogen of the diuron molecule is the initial degradation reaction in fungal metabolism. To our knowledge, this is the first study that demonstrates the involvement of cytochrome P450 in the transformation of diuron and its metabolites by *P. chrysosporium*.

## Figures and Tables

**Table 1:** Effects of 1-aminobenzotriazole on diuron transformation and metabolites production by *P. chrysosporium. *

*P. chrysosporium* culture	Recupered diuron and produced metabolites (*μ*g/mL)					
	5 days			10 days		
	Diuron	DCPMU	DCPU	Diuron	DCPMU	DCPU
Inhibitor free-control	1.72 ± 0.41	0.54 ± 0.20	0.06 ± 0.01	0.26 ± 0.03	0.16 ± 0.06	0.06 ± 0.01
ABT (1 mmol/L)	5.25 ± 0.50	0.22 ± 0.01	0.04 ± 0.01	4.07 ± 0.12	0.32 ± 0.01	0.04 ± 0.02

**Table 2:** Distribution of residual diuron and its metabolites between culture filtrates and mycelial extracts of *P. chrysosporium *after 5 days of cultivation.

Compound	Residual diuron and metabolites after 5 days (*μ*g)		
	Culture filtrate	Mycelial extract	Total
Diuron	71.3 ± 3.3	11.0 ± 0.8	82.3
DCPMU	18.5 ± 0.5	5.6 ± 0.1	24.1
DCPU	1.1 ± 0.1	0.37 ± 0.1	1.5

An amount of 175 *μ*g of diuron was added at zero time in each culture.

**Table 3:** Degradation of diuron by enzymatic crude filtrate from *P. chrysosporium* cultures after 24 h.

Treatment	Veratryl alcohol (0.5 mM)	H_2_O_2_ (0.1 mM)	MnSO_4_ (0.5 mM)	Crude filtrate*	Recupered diuron (*μ*g/mL)
Control	−	−	−	−	6.47 ± 0.43
1	+	+	+	+	7.40 ± 0.46
2	−	+	−	+	6.34 ± 0.72
3	+	+	−	+	5.98 ± 0.35
4	−	+	+	+	6.27 ± 0.31
5	+	−	−	+	5.66 ± 0.48
6^#^	+	+	+	+	7.39 ± 0.39

*40.0 U/L of lignin peroxidase activity and 50 U/L of manganese peroxidase activity. All the treatment contained diuron (7 *μ*g/mL) and sodium malonate buffer 50 mM, pH 4.5. Control was run only with diuron and buffer. ^#^Treatment 6 was performed using boiled crude enzyme. Degradation values are means ± SD (*n* = 3).

**Table 4:** Parameters measured for *L. sativa* bioassay.

	Absolute germination (%)		Relative growth averages (%)			
Sample dilution (v/v)			Radicle		Hypocotyl	
	Abiotic control	10-day treatment	Abiotic control	10-day treatment	Abiotic control	10-day treatment
10%	84.1 ± 2.0	95.0 ± 3.1	42.2 ± 3.7	77.0 ± 4.1*	113.1 ± 7.0	116.2 ± 4.2
20%	80.3 ± 3.6	88.3 ± 2.8*	36.3 ± 8.6	102.6 ± 8.5*	49.8 ± 5.8	122.3 ± 7.3*
50%	78.3 ± 5.7	90.3 ± 5.7*	27.6 ± 2.2	42.8 ± 6.9*	52.2 ± 4.3	88.7 ± 10.5*
80%	58.3 ± 7.6	78.3 ± 2.8*	21.0 ± 1.0	28.4 ± 2.5*	33.4 ± 2.9	65.5 ± 10.4*
100%	31.6 ± 5.7	60.0 ± 7.0*	9.2 ± 1.7	26.8 ± 2.4*	20.8 ± 3.8	44.2 ± 7.2*

The percentage of the absolute germination and the growth averages for lettuce seed bioassays were calculated at five dilutions of nontreated (abiotic control) and 10-day-treated samples, in triplicates. *Significant differences between samples for the same parameter analyzed (*P* < 0.05) by *t*-test.
